# Comparison of Fungal Community in Black Pepper-Vanilla and Vanilla Monoculture Systems Associated with Vanilla *Fusarium* Wilt Disease

**DOI:** 10.3389/fmicb.2016.00117

**Published:** 2016-02-09

**Authors:** Wu Xiong, Qingyun Zhao, Chao Xue, Weibing Xun, Jun Zhao, Huasong Wu, Rong Li, Qirong Shen

**Affiliations:** ^1^National Engineering Research Center for Organic-based Fertilizers, Jiangsu Key Lab for Solid Organic Waste Utilization, Jiangsu Collaborative Innovation Center for Solid Organic Waste Resource Utilization, Nanjing Agricultural UniversityNanjing, China; ^2^Spice and Beverage Research Institute, Chinese Academy of Tropical Agricultural ScienceWanning, China

**Keywords:** continuous cropping, black pepper, soil fungal communities, Miseq sequencing, vanilla healthy growth

## Abstract

Long-term vanilla monocropping often results in the occurrence of vanilla *Fusarium* wilt disease, seriously affecting its production all over the world. In the present study, vanilla exhibited significantly less *Fusarium* wilt disease in the soil of a long-term continuously cropped black pepper orchard. The entire fungal communities of bulk and rhizosphere soils between the black pepper-vanilla system (i.e., vanilla cropped in the soil of a continuously cropped black pepper orchard) and vanilla monoculture system were compared through the deep pyrosequencing. The results showed that the black pepper-vanilla system revealed a significantly higher fungal diversity than the vanilla monoculture system in both bulk and rhizosphere soils. The UniFrac-weighted PCoA analysis revealed significant differences in bulk soil fungal community structures between the two cropping systems, and fungal community structures were seriously affected by the vanilla root system. In summary, the black pepper-vanilla system harbored a lower abundance of *Fusarium oxysporum* in the vanilla rhizosphere soil and increased the putatively plant-beneficial fungal groups such as *Trichoderma* and *Penicillium* genus, which could explain the healthy growth of vanilla in the soil of the long-term continuously cropped black pepper field. Thus, cropping vanilla in the soil of continuously cropped black pepper fields for maintaining the vanilla industry is executable and meaningful as an agro-ecological system.

## Introduction

Vanilla (*Vanilla planifolia*), a herbaceous perennial vine with high economic value, has been widely cropped in tropical and subtropical regions (Minoo et al., 2008). However, the long-term monoculture of this crop often results in the occurrence of soil-borne *Fusarium* wilt disease, seriously affecting its production worldwide in vanilla-cropping regions (Jayasekhar et al., 2008; Pinaria et al., 2010; Xiong et al., 2015b) and leading to significant economic losses over the last decade. Fungicides and biological control agents have been traditionally suggested as integrated control strategies for vanilla *Fusarium* wilt disease (Tombe and Sitepu, 1986; Sandheep et al., 2012); however, these methods are usually environmentally unfriendly or inefficient. Thus, exploring an effective method for controlling the vanilla *Fusarium* wilt disease is extremely important for maintaining the vanilla production. Meanwhile, in tropical China, multiple continuous cropping fields for other tropical crops, such as black pepper and banana, have suffered serious successive cropping obstacles and were given up for growing the same crops (Wang et al., 2013; Xiong et al., 2015a). Because of these associated problems, farmers naturally grow different crops in these fields. By some chance, after our field investigation, an interesting phenomenon was always observed where vanilla with the lowest *Fusarium* wilt disease incidence (DI) grew in the continuously cropped black pepper field. The causes of the disease decline might be very complex, such as improved soil physical and chemical properties and land management practices (Hilton et al., 2013; Navarro-Noya et al., 2013). However, the detailed mechanisms involved in the healthy vanilla growth associated with the soil microflora variation under the soil of long-term continuously cropped black pepper fields remain unclear.

Exploring continuously cropped field soil for other crop growth is meaningful and sustainable to agro-ecological systems. Meanwhile, to our limited knowledge, few studies have focused on the long-term continuously cropped soil supporting other crop growth; thus, how the variation in soil microbiota under long-term continuously cropped soil could support other crop growth is even less well understood. Soil microorganisms play critical roles in regulating soil fertility, global nutrient cycling, and plant health (Fierer et al., 2012), which might be directly linked to the maintenance of plant health in agro-systems. Within soil ecosystems, the immediate surroundings of the plant root, i.e., the rhizosphere, is a dynamic interface supporting the exchange of resources between plants and their associated soil environment (Peiffer et al., 2013). Rhizosphere microbiota, considered as the second genome of the plant, are significantly influenced by plant roots (Philippot et al., 2013). The main source of microbial communities in the rhizosphere is the adjacent root-free soil, called the bulk soil; hence, the changes brought about in the communities of the bulk soil will have an effect on the assembly and the final composition of rhizosphere communities (Mendes et al., 2014).

The development of high-throughput sequencing, particularly Illumina MiSeq sequencing (Metzker, 2010; Shokralla et al., 2012), offers a powerful strategy for uncovering the complex and diverse soil microbial communities with high throughput, high accuracy, and considerably lower cost. The internal transcribed spacers (ITS1) region has been widely used in the analysis of soil fungal communities (Xu et al., 2012; Lu et al., 2013). The functional diversity of soil fungi and their capacity to colonize diverse microhabitats can influence pathogen levels and play a significant role in improving plant health (Penton et al., 2014). Given that vanilla *Fusarium* wilt disease is caused by a fungal pathogen, exploring the fungal community involved in the healthy growth of vanilla in black pepper-vanilla agro-ecosystems is quite important.

Thus, in this study, we hypothesize that long-term continuous cropping black pepper orchards harbored a unique soil fungal community associated with healthy vanilla growth. To test this hypothesis, we used pot experiments to evaluate the persistent ability of the soil of long-term continuously cropped black pepper fields to support vanilla healthy growth; and fungal community of the bulk and rhizosphere soils in the black pepper-vanilla and vanilla monoculture systems was accessed by the Illumina MiSeq sequencing.

## Materials and methods

No specific permits were required for the described field studies. The locations are not protected. The field studied did not involve endangered or protected species.

### Experiment descriptions

The experimental site is located at the Spice and Beverage Research Institute, Wanning City, Hainan Province, China (110°19′E-110°22′E, 18°72′N-18°76′N). It is an area with a tropical monsoon climate, a mean annual temperature of 24.5°C and a mean annual precipitation of 2201 mm. The experimental soil was collected in April 2013 from the 20-years continuously cropped black pepper orchard. The soil was mixed thoroughly and transferred to the greenhouse with an average temperature of 30°C and an average humidity of 72% at the Spice and Beverage Research Institute. Meanwhile, the soil ~200 m away from the black pepper orchard collected from the 21-year continuously cropped vanilla orchard showing serious vanilla *Fusarium* wilt disease (Xiong et al., 2015b) was considered a control. Both the black pepper and vanilla orchard soils are sandy loam in texture and developed from the same parent material. The experiment was performed using a randomized complete block design in three replicates, where each block had six pots for each treatment, and each pot contained 15 kg soil with three seedling vanillas. The agronomic management and fertilization regime were uniform during the next 18 months (April, 2013 to October, 2014). Vanilla *Fusarium* wilt disease was monitored immediately after the seedlings were transplanted into the pots based on the observation of typical wilt symptoms. The DI was calculated as the percentage of infected plants among the total number of plants (Wei et al., 2011). It is worth noting that we also used continuously cropped banana and coffee orchards soil to cultivate vanilla in pots. We found the continuously cropped black pepper soil showed the lowest vanilla *Fusarium* wilt disease and the highest plant biomass (vanilla shoot dry weight). Hence, we got the two vanilla cropping regimes, i.e., black pepper-vanilla system and vanilla monoculture system for the subsequent research.

### Soil sample collection and DNA extraction

After removing the vanilla plants from the pots, the bulk soil samples obtained for each replicate from the black pepper-vanilla and vanilla monoculture systems were referred to as “BB” and “VB,” respectively. All six bulk soil samples were passed through a 2 mm sieve, thoroughly homogenized and divided into 2 subsamples: one was air-dried for a soil characteristic analysis according to our previous methods (Xiong et al., 2015b), and the remainder was stored at −80°C for DNA extraction. For the vanilla rhizosphere soil samples, six vanilla plants were randomly selected from each replicate in the black pepper-vanilla and vanilla monoculture systems, the roots were vigorously shaken to dislodge the loosely adhering soil, and the soil remaining attached to the root system was considered to be rhizosphere soil. The rhizosphere soil was collected using the following protocol: the roots were cut into pieces of ~1 cm length and carefully mixed, 20 g of roots were pooled into a 500 mL vol. flask containing 200 mL ddH_2_O and washed on a shaking platform for 20 min at 180 rpm, the washing buffer was subjected to centrifugation (10,000 g, 10 min), and then the resulting pellet was obtained and defined as the rhizosphere soil. The rhizosphere soil from the black pepper-vanilla system and vanilla monoculture system are referred to as “BR” and “VR,” respectively. All six rhizosphere soil samples were stored at −80°C for DNA extraction.

Total DNA was extracted from the 12 soil samples using a MoBioPowerSoil™ DNA Isolation Kit (Mo Bio Laboratories Inc., Carlsbad, CA, USA) according to the manufacturer's instructions. The genomic DNA concentration and purity were measured using NanoDrop ND-2000 (NanoDrop Technologies, Wilmington, DE) spectrophotometry.

### PCR amplification and deep pyrosequencing

The fungi-specific primers ITS1F (CTTGGTCATTTAGAGGAAGTAA) (Gardes and Bruns, 1993) and ITS2 (GCTGCGTTCTTCATCGATGC) (White et al., 1990) were selected to target the ITS1 region. These primer pairs were modified for pyrosequencing by adding the forward Illumina Nextera adapter, a two-base-pair “linker” sequence, and a unique 7-bp barcode sequence to the 5′ end of the forward primer and the appropriate reverse Illumina Nextera adapter and linker sequence at the 5′ end of the reverse primer. PCR amplification was performed in a 25 μl reaction: 2.5 μl of 10 × reaction buffer, 10 μM of each primer, 2.5 mM dNTPs, 40 ng of template, and 0.625 units of Takara Pyrobest (Takara Biotechnology Co., Ltd., Japan). Amplifications were performed with the following temperature regime: 4 min of initial denaturation at 94°C, followed by 35 cycles of denaturation (94°C for 30 s), annealing (50°C for 45 s), extension (72°C for 1 min), and a final extension at 72°C for 7 min. The PCR products were purified using a PCR Purification Kit (Axygen Bio, USA). Then, paired-end sequencing was performed on an Illumina MiSeq sequencer at Personal Biotechnology Co., Ltd (Shanghai, China).

### Quantification of the *Fusarium oxysporum* and fungal abundances

Real-time quantitative polymerase chain reaction (qPCR) was performed according to Chen et al. (2014) for quantifying the soil *Fusarium oxysporum* and fungi abundances using the SYBR Premix Ex Taq Kit on the ABI PRISM 7500 Real Time PCR System (Applied Biosystems, Germany). The 20 μl reaction mixture contained 10 μl of the *Premix Ex Taq*™ (2 ×) (Takara), 0.4 μl of each primer (10 μM), 0.4 μl of ROX Reference Dye II (50 ×), 2 μl of template DNA, and 6.8 μl of ddH_2_O. The specific primer set of *F. oxysporum* and soil fungi was AFP308R (CGAATT AACGCGAGTCCCAAC)/ITS1F (CTTGGTCATTTAGAGGAAGTAA) (Lievens et al., 2006) and ITS2 (GCTGCGTTCTTCATCGATGC)/ITS1F (CTTGGTCATTTA GAGGAAGTAA), respectively. The thermal conditions were set as follows: 30 s at 95°C for initial denaturation, 40 cycles of 5 s at 95°C, and 34 s at 60°C. The standard curve was obtained using a 10-fold dilution series of plasmid DNA containing a fragment of the ITS region of *F. oxysporum* and ITS1 gene from the *F. oxysporum* f. sp. *vanillae* and soil samples, respectively. All amplifications were performed in triplicate. The specificity of the products was confirmed by a melting curve analysis and agarose gel electrophoresis. The copy numbers were log_10_-transformed to normalize the values prior to statistical analysis.

### Pyrosequencing data analysis

After removing the adaptors and primer sequences, the raw sequences were assembled for each sample according to the unique barcode using QIIME (Caporaso et al., 2010). The split sequences for each sample were merged using FLASH V1.2.7 (Magoč and Salzberg, 2011), and low-quality sequences were then discarded using QIIME. The sequences retained for each sample were processed following the established UPARSE pipeline (Edgar, 2013). Briefly, the sequences with a quality score lower than 0.5 or a length shorter than 200 bp were removed. After discarding the singletons, the remaining reads were assigned to OTUs with a threshold of 97% identity level. Then, the chimera removal processes were performed. Finally, the fungal representative OTUs were classified using the UNITE database (Kõljalg et al., 2013).

The diversity within each individual sample was estimated using non-parametric Shannon diversity indices. Shannoneven was used to measure the evenness of each sample (Schloss et al., 2009). A principal coordinate analysis (PCoA) based on weighted UniFrac metric matrices was performed to explore the differences in fungal community structures among all of the soil samples (Lozupone et al., 2006). A permutational multivariate analysis of variance (Anderson, 2001) was performed to assess the effect of the cropping regime, soil compartment, and their interactions on the fungal community structure (abundance of OTUs and genus) using the adonis function of the R vegan package with 999 permutations.

### Statistical analyses

The soil physicochemical characteristics and vanilla *Fusarium* wilt DI between the black pepper-vanilla and vanilla monoculture systems were compared using Student's *t*-test. For other parameters in our study, one-way analyses of variance (ANOVA) with Turkey's HSD multiple range test were performed for multiple comparisons. All of the statistical analyses were performed using SPSS v20.0 (SPSS Inc., USA).

### Sequence accession numbers

The sequence data have been deposited in the NCBI Sequence Read Archive (SRA) database with the accession number SRP062990.

## Results

### Soil physical and chemical properties in the black pepper-vanilla and vanilla monoculture systems

The results of soil physical and chemical properties are summarized in Table S1. When compared with the vanilla monoculture system, black pepper-vanilla system presented a significantly (*P* < 0.05) higher available N content. In contrast, the vanilla monoculture system revealed higher soil pH and the contents of organic matter and available P.

### *Fusarium* wilt DI and fungal abundance in the two vanilla cropping systems

As shown in Table 1, the black pepper-vanilla system significantly reduced vanilla *Fusarium* wilt DI to 15.56%, whereas the value was over 60% in the vanilla monoculture system. The qPCR results showed that the ITS copies of *F. oxysporum* were significantly lower in the black pepper-vanilla system compared with those from the vanilla monoculture system in both bulk and rhizosphere soils (Table 1). Strikingly, the *F. oxysporum* populations significantly increased from the bulk soil to the vanilla rhizosphere soil in both the black pepper-vanilla and vanilla monoculture systems. In addition, the fungal ITS gene copy numbers in bulk soil showed no significant difference between the black pepper-vanilla and vanilla monoculture systems (Table S2). Meanwhile, the fungal ITS gene copy numbers in the rhizosphere soil samples from the black pepper-vanilla system (8.15 × 10^9^ copies g^−1^ soil) were significantly higher than those from the vanilla monoculture system (2.89 × 10^9^ copies g^−1^ soil).

**Table 1:** **Vanilla *Fusarium* wilt disease incidence and pathogen abundance**.

**Cropping regime**	**Soil compartment**	**Disease incidence (%)**	***Fusarium* RA**	***F. oxysporum* RA**	**Log_10_*F. oxysporum* ITS copies g^−1^ soil**
Black pepper-vanilla system	Bulk soil (BB)	15.56 ± 3.85 b	6.79±2.23 b	5.51±2.75 b	4.80±0.08 d
	Rhizosphere soil (BR)		10.36±1.31 b	8.66±1.15 b	5.60±0.15 b
Vanilla monoculture system	Bulk soil (VB)	62.22 ± 10.08 a	10.23±0.82 b	5.59±0.37 b	5.13±0.13 c
	Rhizosphere soil (VR)		26.18±7.54 a	22.89±6.80 a	6.18±0.09 a

*Values are means ± standard deviation (n = 3). RA, Relative abundance*.

*Means followed by the same letter for a given factor are not significantly different (P < 0.05; Turkey's HSD test where there are more than two treatment levels and Student's t-test where there are two treatment levels)*.

### Overall diversity of fungal community

**Figure 1:**
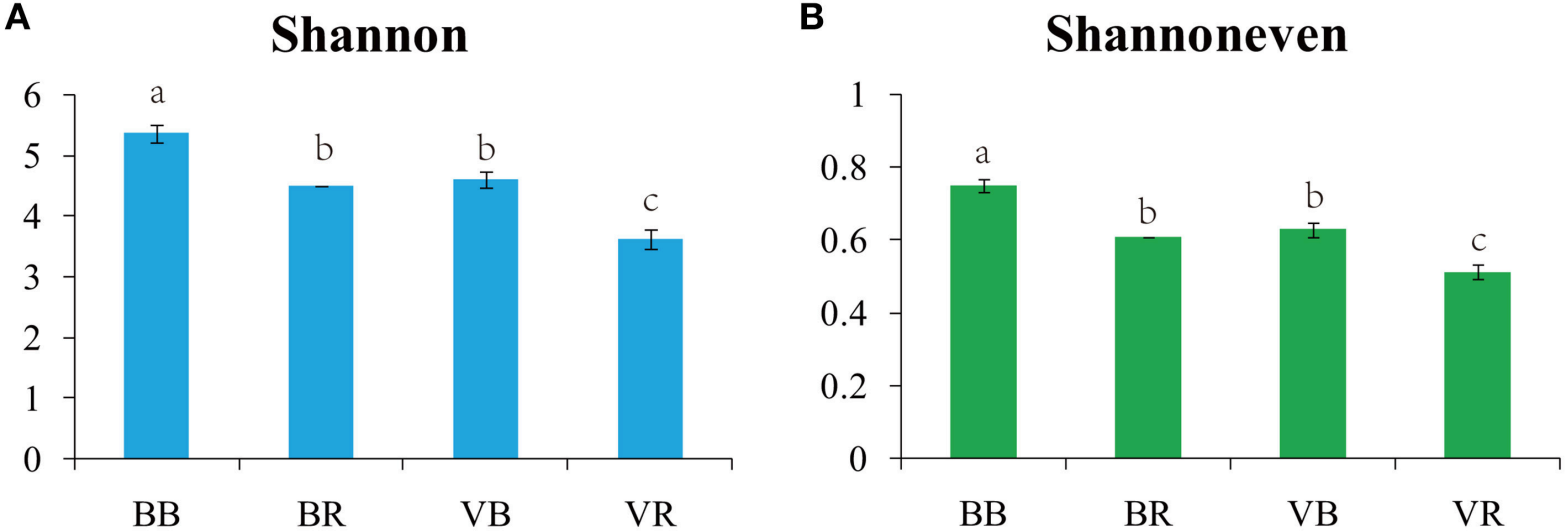
**Shannon diversity indices (A) and Shannoneven indices (B) for the bulk soil and vanilla rhizosphere soil of the black pepper-vanilla and vanilla monoculture systems**. Bars represent the standard deviation of the three replicates, and different letters above the bars indicate a significant difference at the 0.05 probability level according to the Turkey's HSD test. “BB” and “VB” represent the bulk soil from the black pepper-vanilla system and the vanilla monoculture system, respectively. “BR” and “VR” represent the rhizosphere soil from the black pepper-vanilla system and the vanilla monoculture system, respectively.

After quality filtering, the pyrosequencing-based analysis of the fungal ITS1 genes resulted in the recovery of 1,260,032 high-quality sequences across the 12 samples (Table S3). The coverage from all samples was above 99%, indicating that the sequencing reads were sufficient for this analysis (Table S3). In both the bulk and rhizosphere soils, the black pepper-vanilla system had a significantly higher fungal diversity (Shannon) and evenness (Shannoneven) values than the vanilla monoculture system (Figure 1). In addition, in both the black pepper-vanilla and vanilla monoculture systems, the fungal community diversity and evenness significantly decreased from the bulk soil to the vanilla rhizosphere soil.

### Fungal community composition

**Figure 2:**
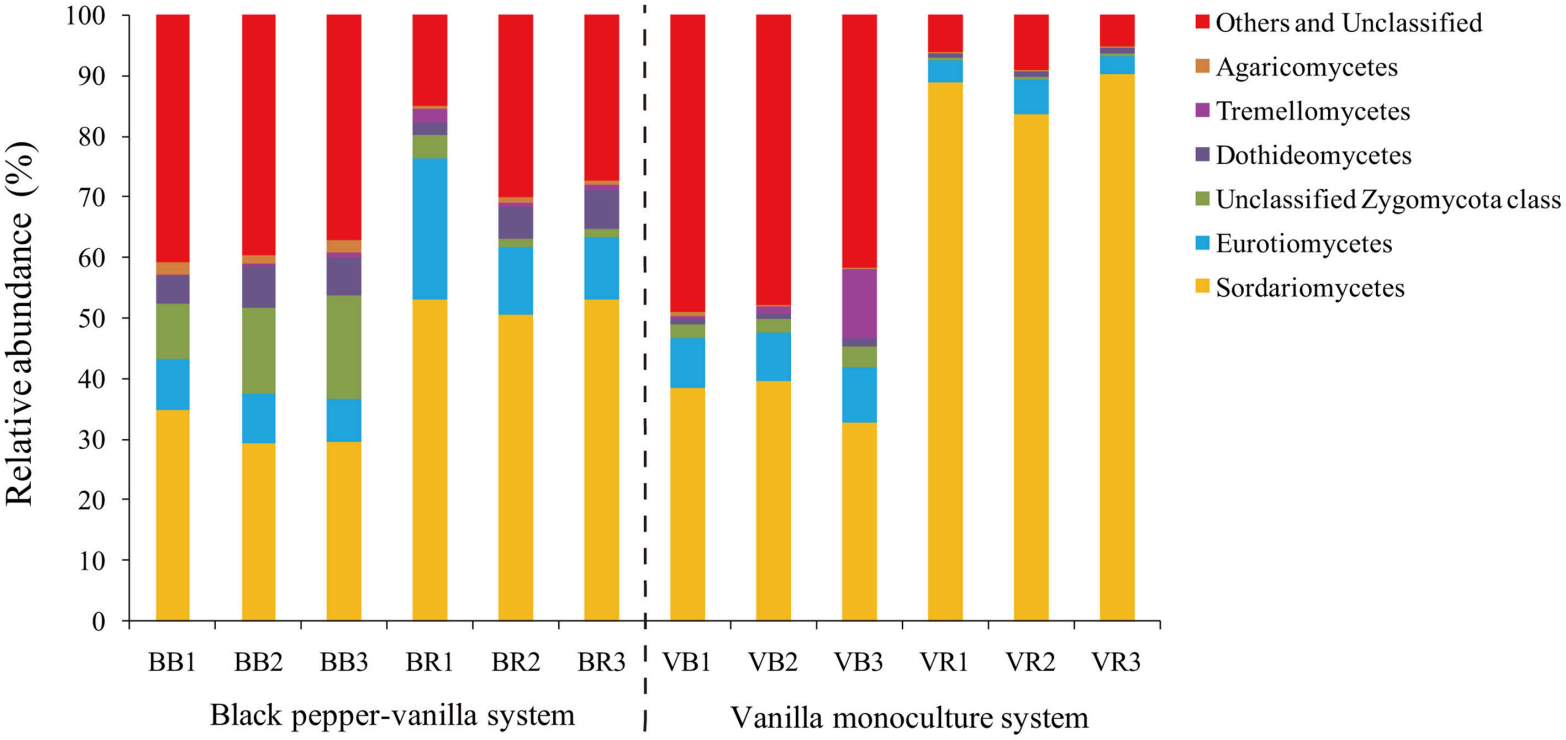
**Relative abundances of the main fungal classes in the bulk soil and the vanilla rhizosphere soil of the black pepper-vanilla and vanilla monoculture systems**. The “Others and Unclassified” comprised the unclassified and low-abundance classes (RA < 0.1%). “BB” and “VB” represent the bulk soil from the black pepper-vanilla system and the vanilla monoculture system, respectively. “BR” and “VR” represent the rhizosphere soil from the black pepper-vanilla system and the vanilla monoculture system, respectively.

**Figure 3:**
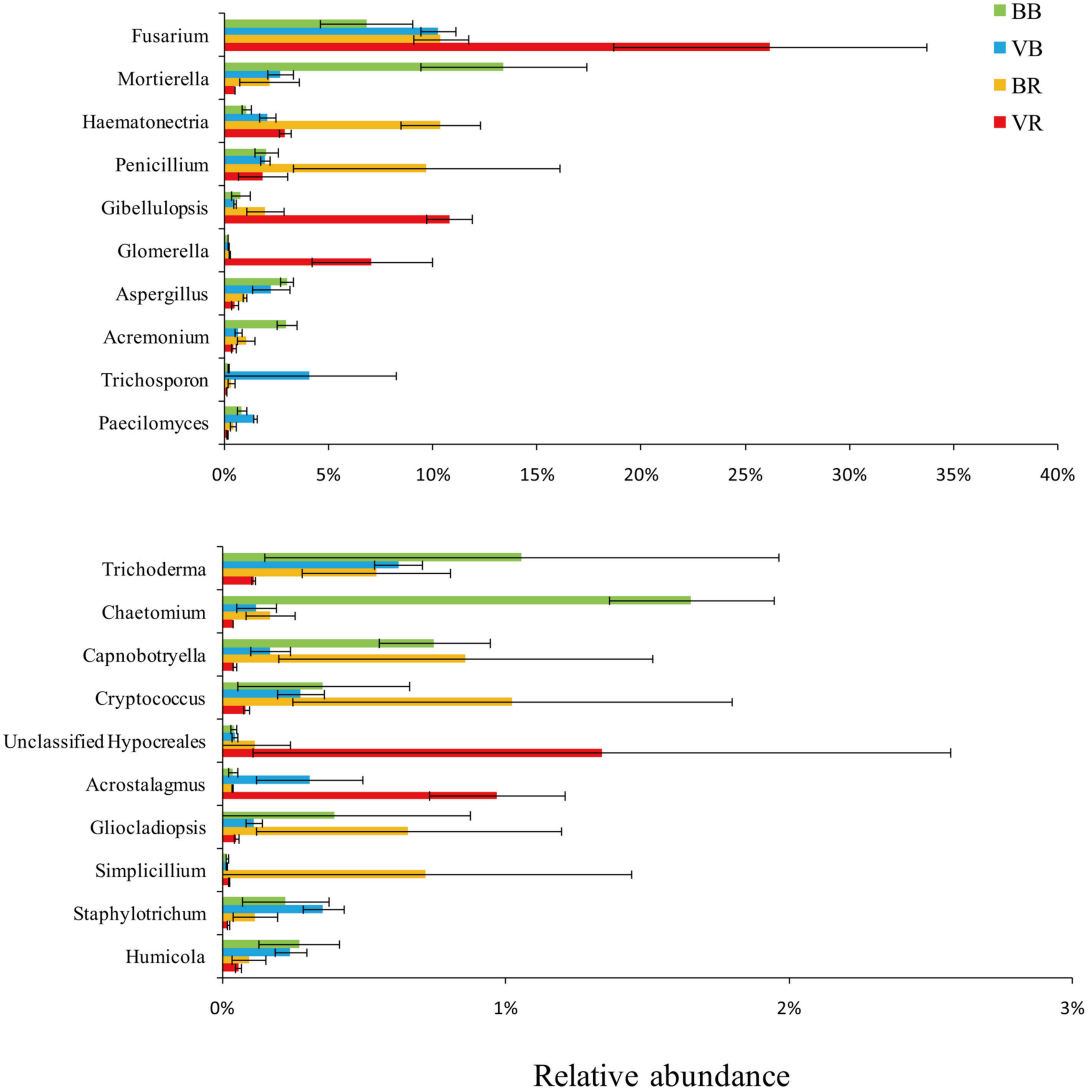
**Relative abundances of the top 20 fungal genera in bulk soil and vanilla rhizosphere soil of the black pepper-vanilla and vanilla monoculture systems**. Bars represent the standard deviation of the three replicates. “BB” and “VB” represent the bulk soil from the black pepper-vanilla system and the vanilla monoculture system, respectively. “BR” and “VR” represent the rhizosphere soil from the black pepper-vanilla system and the vanilla monoculture system, respectively.

To verify the differences observed in the fungal communities from the black pepper-vanilla and vanilla monoculture systems, the relative abundances (RA) of the different classes and genera from the bulk and rhizosphere soils were compared (Figures 2, 3). In the present study, fungal OTUs across the 12 soil samples were observed predominantly from the six classes (*Sordariomycetes, Eurotiomycetes*, unclassified *Zygomycota* class, *Dothideomycetes, Tremellomycetes*, and *Agaricomycetes*), accounting for 71.10% of the total fungal sequences. Compared with bulk soil, the relative abundance of the class *Sordariomycetes* in the vanilla rhizosphere soil significantly increased in both the black pepper-vanilla and vanilla monoculture systems. At the genus level, in the bulk soil, compared with the vanilla monoculture system, the black pepper-vanilla system had a higher relative abundance of *Mortierella, Aspergillus, Acremonium*, and *Chaetomium*. As for the rhizosphere soil, the *Fusarium* genus was significantly more abundant in the vanilla monoculture system than in the black pepper-vanilla system; moreover, the relative abundance of *F. oxysporum* (OTU level) exhibited a similar trend (Table 1). In addition, the relative abundances of the genera *Haematonectria, Trichoderma*, and *Penicillium* were significantly higher in the black pepper-vanilla system with a lower *Gibellulopsis* abundance.

### Fungal community structure

A permutational multivariate analysis of variance confirmed that the cropping regime, soil compartment, and their interactions were significant factors of variation for the fungal community structure in terms of both the relative abundance of OTUs and relative abundance of genera (Table 2).

**Table 2:** **PERMANOVA analysis**.

**Source**	**Df**	**Abundance of Genera**		**Abundance of OTUs**	
		**Sums of sqs**	**Pseudo-F**	**Sums of sqs**	**Pseudo-F**
Cropping regime (CR)	1	0.18	11.79^***^	0.42	7.64^***^
Soil compartment (SC)	1	0.25	15.80^***^	0.40	7.30^***^
CR × SC	1	0.09	5.54^***^	0.20	3.68^***^
Residuals	8	0.13	0.19	0.44	0.30

*** *Indicate significant correlations (P < 0.001)*.

**Figure 4:**
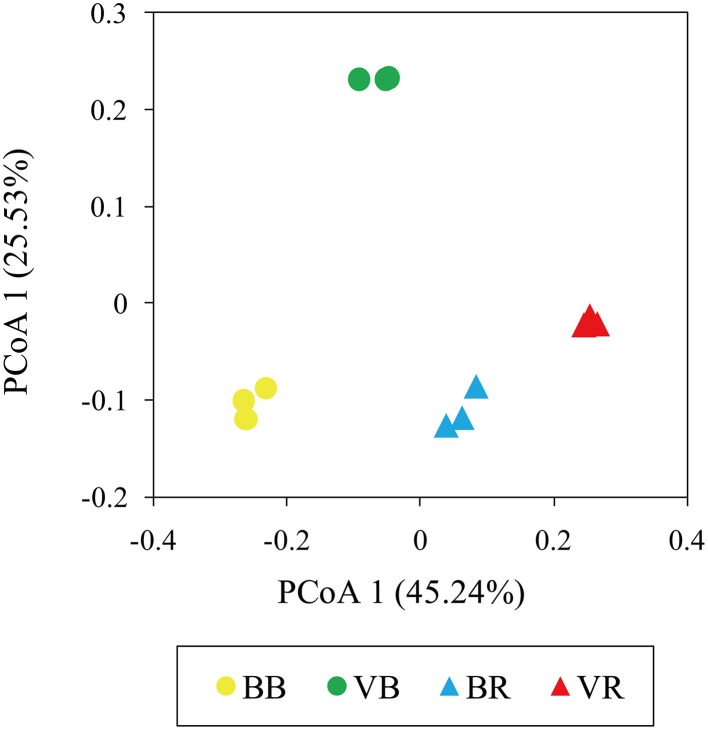
**UniFrac-weighted principle coordinate analysis of fungal community structures in the bulk soil and vanilla rhizosphere soil of the black pepper-vanilla and vanilla monoculture systems**. “BB” and “VB” represent the bulk soil from the black pepper-vanilla system and the vanilla monoculture system, respectively. “BR” and “VR” represent the rhizosphere soil from the black pepper-vanilla system and the vanilla monoculture system, respectively.

To further compare the variations in fungal community structure between the black pepper-vanilla and vanilla monoculture systems samples, a UniFrac-weighted PCoA was employed. As shown in Figure 4, the bulk soil samples from the black pepper-vanilla system were clearly separated from the vanilla monoculture system, suggesting strong differences in fungal community structures between the different crop regime systems. In addition, the fungal communities in the rhizosphere soils from the two vanilla cropping systems were close together, suggesting that fungal community structures were seriously affected by the vanilla root system.

## Discussion

Obstacles to the continuous cropping of vanilla have always been observed on Hainan Island (Xiong et al., 2015b). In the present study, pot experiments confirmed that long-term continuously cropped black pepper orchard soil showed significantly lower vanilla *Fusarium* wilt disease, implying that crop rotation is an effective management practice to reduce soil-borne plant disease in agro-systems (Wang et al., 2015). In addition, it will also help us to take advantage of the large area of black pepper continuous cropping soil in tropical China (Zu et al., 2014; Xiong et al., 2015a).

In this study, the black pepper-vanilla system had no effect on the fungal population abundance in the bulk soil. However, alpha diversity estimates of the fungal communities revealed that the black pepper-vanilla system had a significantly higher fungal diversity and evenness than the vanilla monoculture soil (Figure 1). The possible reasons are as follows: residues of black pepper decomposed in the soil and different root exudates could provide more available nutrient to soil microbes, thus improving species richness, heterogeneity, and diversity of the fungal community (Xuan et al., 2011). Furthermore, we could find a negative relationship between the soil fungal diversity and vanilla *Fusarium* wilt disease, which could support the idea that microbial diversity is a key factor in controlling pathogen invasion (van Elsas et al., 2012). In addition, in the present study, the fungal community diversity significantly decreased from the bulk soil to the vanilla rhizosphere soil in both the black pepper-vanilla and vanilla monoculture systems; as explained by Mendes et al. (2014) plants can select a constant rhizosphere community from highly contrasting reservoirs of bulk soil communities.

The UniFrac-weighted PCoA analysis revealed significant variations in the bulk soil of fungal community structures between the black pepper-vanilla and vanilla monoculture systems (Figure 4). Our results agreed with the findings of Wang et al. (2015) where the crop regime system was the major determinant factor for microbial community structures. The significant variations in bulk soil community structures among the different cropping systems might be attributed to significant differentiations in soil physicochemical characteristics (Table S1), as soil physicochemical properties have significant impacts on microbial community structures (Lauber et al., 2008). Compared with the vanilla monoculture system, black pepper-vanilla system revealed a significantly higher available N content, as nitrogen play a pivotal role in plant growth and might indirectly enhance plant disease suppressiveness (Hayat et al., 2010). In addition, the UniFrac-weighted PCoA analysis suggested fungal community structures were also seriously affected by the vanilla root system, which was consistent with the many previous studies that plant play a key role in shaping the microbial community structures in the rhizosphere (Philippot et al., 2013; Edwards et al., 2015).

The black pepper-vanilla system was shown to have a significant effect on the fungal community compositions in both bulk and rhizosphere soils. *Sordariomycetes* was the most abundant fungal class (Figure 2), which was generally consistent with the many early studies that found *Sordariomycetes* to be the most common fungal class in different agricultural systems (Chen et al., 2012; Li et al., 2014). Compared with bulk soil, the abundance of *Sordariomycetes* significantly increased in vanilla rhizosphere soil in both the black pepper-vanilla and vanilla monoculture systems, as reported by Zhang et al. (2006) who found that members of the *Sordariomycetes* are ubiquitous in virtually all ecosystems as pathogens and endophytes of plants.

Deeper taxonomic analyses were performed to explore the fungal community compositions of rhizosphere soil in the black pepper-vanilla system associated with vanilla growth. The black pepper-vanilla system showed significantly lower *Fusarium* and *F. oxysporum* abundance in the vanilla rhizosphere soil, which could be the most important reason for significantly lower vanilla *Fusarium* wilt disease in the black pepper-vanilla system (Pinaria et al., 2010). The *F. oxysporum* abundance is lower in the black pepper-vanilla system might be because that continuous cropping black pepper soil had not previously been used to cultivate vanilla, however, if vanilla is continuously cropped in that soil which could also increase the pathogen load eventually (Xiong et al., 2015b). In addition, in present study, the *F. oxysporum* populations significantly accumulated from the bulk soil to the vanilla rhizosphere soil in both the black pepper-vanilla and vanilla monoculture systems. Synthesized from the above results, we conclude that iterative crop rotation might be necessary to interrupt the accumulation of *F. oxysporum* abundance to suppress vanilla *Fusarium* wilt disease.

Some putatively plant-beneficial fungal groups, such as the genera *Trichoderma* and *Penicillium*, increased in the vanilla rhizosphere soil under the black pepper-vanilla system. *Trichoderma* spp. are known to have an effective antagonistic effect against vanilla *Fusarium* wilt disease (Jayasekhar et al., 2008; Vijayan et al., 2009). *Penicillium* is also a famous biocontrol agent for the biological control of *Fusarium* wilt disease (Larena et al., 2003); however, this has not yet been reported in vanilla systems. Moreover, *Haematonectria* was the most abundant genus, accounting for 10.33% of the total fungal genera in vanilla rhizosphere soil in the black pepper-vanilla system, which could occupy the rhizosphere niche to avoid pathogen invasion (Qiu et al., 2013). Combining the other variations in fungal genera in the black pepper-vanilla and vanilla monoculture systems and the complex interactions among these microorganisms could explain the status of vanilla *Fusarium* wilt disease in agro-ecosystems.

In conclusion, compared with the vanilla monoculture system, black pepper-vanilla system harbored a significantly lower abundance of *F. oxysporum* in vanilla rhizosphere soil, increased the putatively plant-beneficial fungal groups and the fungal diversity, which could explain the decrease in vanilla *Fusarium* wilt disease in the soil of the long-term continuously cropped black pepper orchard. These results suggested that sustainable agricultural management regime, such as crop rotation might be a meaningful strategy to prevent vanilla *Fusarium* wilt disease occurrence and will be our future research focus.

## Author contributions

Conceived and designed the experiments: W. Xiong, QZ, RL, HW, QS. Performed the experiments: W. Xiong, QZ, CX, W. Xun, JZ. Analyzed the data: W. Xiong, QZ, CX, JZ. Contributed reagents/materials/analysis tools: W. Xun, RL, HW, QS. Wrote the manuscript: W. Xiong, RL, HW, QS.

### Conflict of interest statement

The authors declare that the research was conducted in the absence of any commercial or financial relationships that could be construed as a potential conflict of interest.
