# Supplementary material for: Comparison of Fungal Community in Black Pepper-Vanilla and Vanilla Monoculture Systems Associated with Vanilla Fusarium Wilt Disease
Source: Front Microbiol. 2016 Feb 9;7:117. doi: 10.3389/fmicb.2016.00117 (PMC4746283; doi:10.3389/fmicb.2016.00117)
Supplement: Supplementary file 1 [file Table1.DOCX]

**Table S1** Soil physicochemical characteristics.

| Field sites | pH | EC (μs/cm) | Organic matter (g/kg) | Available N (mg/kg) | Available P (mg/kg) | Available K (mg/kg) |
| --- | --- | --- | --- | --- | --- | --- |
| Black pepper-vanilla  system | 5.63±0.24 b | 313.33±30.99 b | 17.65±1.27 b | 141.61±4.83 a | 201.90±14.81 b | 201.34 ± 16.83 a |
| Vanilla monoculture system | 6.23±0.14 a | 392.33±27.75 a | 24.77±1.13 a | 93.30±9.18 b | 321.25±14.68 a | 188.29±7.23 a |

Values are means ± standard deviation (n = 3).

Means followed by the same letter for a given factor are not significantly different (*P* < 0.05; Student's t-test).
