# Supplementary material for: Comparison of Fungal Community in Black Pepper-Vanilla and Vanilla Monoculture Systems Associated with Vanilla Fusarium Wilt Disease
Source: Front Microbiol. 2016 Feb 9;7:117. doi: 10.3389/fmicb.2016.00117 (PMC4746283; doi:10.3389/fmicb.2016.00117)
Supplement: Supplementary file 2 [file Table2.DOCX]

**Table S2** Fungal abundance.

| Cropping regime | Soil compartment | Log_10_ ITS gene copies g^−1^ soil |
| --- | --- | --- |
| Black pepper-vanilla  system | Bulk soil (BB) | 8.63±0.18 c |
|  | Rhizosphere soil (BR) | 9.91±0.01 a |
| Vanilla monoculture system | Bulk soil (VB) | 8.72±0.20 c |
|  | Rhizosphere soil (VR) | 9.46±0.04 b |

Values are means ± standard deviation (n=3).

Means followed by the same letter for a given factor are not significantly different (*P* < 0.05; Turkey’s HSD test).
