# Supplementary material for: Comparison of Fungal Community in Black Pepper-Vanilla and Vanilla Monoculture Systems Associated with Vanilla Fusarium Wilt Disease
Source: Front Microbiol. 2016 Feb 9;7:117. doi: 10.3389/fmicb.2016.00117 (PMC4746283; doi:10.3389/fmicb.2016.00117)
Supplement: Supplementary file 3 [file Table3.DOCX]

**Table S3** Average good quality sequences and coverages for each treatment.

| Cropping regime | Soil compartment | Good quality sequences | Coverage (%) |
| --- | --- | --- | --- |
| Black pepper-vanilla  system | Bulk soil (BB) | 99431±15698 ab | 99.44±0.01 c |
|  | Rhizosphere soil (BR) | 89801±4483 b | 99.56±0.04 b |
| Vanilla monoculture system | Bulk soil (VB) | 114151±3093 a | 99.52±0.02 b |
|  | Rhizosphere soil (VR) | 116627±2670 a | 99.63±0.03 a |

Values are means ± standard deviation (n=3).

Means followed by the same letter for a given factor are not significantly different (*P* < 0.05; Turkey’s HSD test).
